# Varicella Zoster Virus (VZV)-Human Neuron Interaction

**DOI:** 10.3390/v5092106

**Published:** 2013-09-04

**Authors:** Nicholas L. Baird, Xiaoli Yu, Randall J. Cohrs, Don Gilden

**Affiliations:** 1 Departments of Neurology, University of Colorado School of Medicine, Aurora, CO 80045, USA; E-Mails: nicholas.baird@ucdenver.edu (N.L.B.); xiaoli.yu@ucdenver.edu (X.Y.); randall.cohrs@ucdenver.edu (R.J.C.); 2 Departments of Microbiology, University of Colorado School of Medicine, Aurora, CO 80045, USA

**Keywords:** varicella zoster virus, neurons, latency

## Abstract

Varicella zoster virus (VZV) is a highly neurotropic, exclusively human herpesvirus. Primary infection causes varicella (chickenpox), wherein VZV replicates in multiple organs, particularly the skin. Widespread infection *in vivo* is confirmed by the ability of VZV to kill tissue culture cells *in vitro* derived from any organ. After varicella, VZV becomes latent in ganglionic neurons along the entire neuraxis. During latency, virus DNA replication stops, transcription is restricted, and no progeny virions are produced, indicating a unique virus-cell (neuron) relationship. VZV reactivation produces zoster (shingles), often complicated by serious neurological and ocular disorders. The molecular trigger(s) for reactivation, and thus the identity of a potential target to prevent it, remains unknown due to an incomplete understanding of the VZV-neuron interaction. While no *in vitro* system has yet recapitulated the findings in latently infected ganglia, recent studies show that VZV infection of human neurons in SCID mice and of human stem cells, including induced human pluripotent stem cells and normal human neural progenitor tissue-like assemblies, can be established in the absence of a cytopathic effect. Usefulness of these systems in discovering the mechanisms underlying reactivation awaits analyses of VZV-infected, highly pure (>90%), terminally differentiated human neurons capable of prolonged survival *in vitro.*

## 1. Introduction

Primary infection with varicella zoster virus (VZV), a highly neurotropic, exclusively human herpesvirus, causes varicella (chickenpox), during which time VZV replicates in multiple organs, particularly the skin [1]. After varicella, VZV remains latent in neurons of cranial nerve ganglia, dorsal root ganglia and autonomic ganglia along the entire neuraxis [2,3,4]. In contrast to productive infection where virus DNA replication, transcription and translation of the ~70 VZV genes are robust, latent infection of neurons is unique in that virus DNA does not replicate and transcription is restricted to <12 virus genes, and no progeny virions are produced [5]. Upon VZV reactivation, new virions are assembled and transported anterograde to infect dermal cells, resulting in zoster (shingles). Zoster is characterized by dermatomal distribution rash and pain. Pain often becomes chronic (postherpetic neuralgia). After reactivation, the virus may also travel retrograde and produce meningoencephalitis, cranial nerve palsies, vasculopathy, myelopathy, and various inflammatory disorders of the eye [6]. Because VZV becomes latent in and reactivates from neurons exclusively, numerous attempts have been made to study VZV-neuronal interactions *in vivo* and *in vitro*. VZV has been used to infect: (1) cultures of human ganglia, (2) human neural cells maintained as xenografts in mice, and, most recently, (3) embryonic stem (ES) cells and induced pluripotent stem (iPS) cells. The ultimate development of a model in which VZV becomes latent in neurons, characterized by the absence of a virus-induced cytopathic effect (CPE) and limited VZV transcription will provide a system to study molecules that induce reactivation. Identification of such molecules promises to identify therapeutic targets to prevent virus reactivation, a cause of serious, sometimes fatal neurologic disease, especially in the elderly/immunocompromised populations. This review presents an overview of VZV configuration and viral gene expression in latently infected human ganglia, followed by a comparison of various model systems designed to produce an equivalent virus-host relationship in neurons *in vitro*. 

## 2. Latent VZV in Human Ganglia

During varicella, productive VZV infection occurs not only in the skin, but also in visceral organs. Widespread infection *in vivo* is confirmed by the ability of VZV to kill tissue culture cells *in vitro* derived from any organ. After varicella, VZV becomes latent in ganglionic neurons. Unlike primary measles virus infection of humans, varicella infection does not appear to involve the brain and spinal cord, a notion supported by the absence of simian varicella virus (SVV) in the CNS of non-human primates [7]. During latency, the VZV genomic termini join to form an “endless” molecule [8], with ganglia containing 35–3500 copies of VZV DNA per 100 ng ganglionic DNA [9,10,11]. Although the full extent of VZV transcription during latency is unknown, RT-PCR and *in situ* hybridization studies have identified multiple VZV transcripts in latently infected human ganglia. State-of-the-art multiplex PCR technology, capable of detecting all 68 annotated VZV gene transcripts, revealed transcription of at least 12 VZV genes during latency [12,13], of which VZV open reading frame (ORF) 63 transcripts are the most prevalent and abundant [14]. Importantly, autopsy in the first 9 h after death reveals transcription only of VZV ORF 63 [15]. Promoters for VZV ORFs 62 and 63 are associated with histone protein 3 acetylated on lysine 9, a post-translational modification indicative of active transcription [16]. Immunohistochemical analysis has identified some virus-specific proteins in latently infected human ganglia [5], results that are discordant with other data, possibly due to cross-reactivity of various anti-VZV antibodies with human blood group A determinants in sensory neurons [17], thus requiring confirmation by independent techniques.

## 3. Explanted Human Ganglia

The first *in vitro* studies of the VZV-neuron relationship used VZV infection of explanted human brain and ganglia cells. Because most cells in these cultures were not neurons, VZV infection was productive. Interestingly, large intracytoplasmic vacuoles were seen in VZV-infected brain and ganglionic cultures, but not in control VZV-infected fibroblasts [18]. Human fetal dorsal root ganglion (DRG) infected with cell-associated and cell-free VZV showed the presence of viral proteins by immunofluorescence and of viral particles by electron microscopy in neurons [19]. Once again, a CPE developed, although neurons were thought to be less susceptible than non-neuronal cells to a lytic effect. Subsequently, cocultivation of dissociated human fetal DRG with VZV-infected fibroblasts led to productive infection of neurons 2 days post-infection (d.p.i.); although neurons were not examined for virus weeks after infection, they were shown to be resistant to apoptosis [20]. Further studies of human fetal DRG explants containing both neurons and non-neuronal cells infected with VZV revealed productive infection, with cell-free virus titers peaking at 4 d.p.i and sharply declining by day 5 [21]. A recent study of human trigeminal ganglia latently infected with VZV and HSV and maintained in culture after collection at autopsy [22] showed that after explantation, VZV DNA copy number increased over time but that HSV DNA increased 4-fold more than VZV DNA, possibly explaining the inability to rescue VZV from latently infected human ganglia.

## 4. Human Neurons in SCID Mice

Human neural stem cells isolated from fetal brain remain viable and are capable of differentiation after transplantation into brains of non-obese, diabetic, severe combined immunodeficient (SCID) mice [23]. At 4–6 months after transplantation, VZV-infected human melanoma cells were directly injected into the lateral ventricles of SCID mice; 3 weeks later, VZV immediate-early (IE) proteins 62 and 63 and the VZV early protein 47 were detected in the transplanted neuronal tissue, while the VZV late gene product gE was only rarely found. Subsequently, human fetal DRGs were implanted under the kidney capsule of SCID mice [24]. After 4–12 weeks, VZV-infected fibroblasts were injected into the xenografts. Two weeks after infection, the DRGs had detectable viral DNA and VZV transcripts, and infectious virus was recovered when the xenografts were cocultivated with uninfected fibroblasts; ultrastructural analysis of the DRGs revealed virions, although most of the virus particles lacked electron-dense cores and envelopes. Four weeks after infection, the virus was not seen ultrastructually, infectious VZV could not be recovered, and VZV DNA and VZV-specific transcripts were less abundant, although the transcripts examined were detectable. At 8 weeks p.i., VZV DNA copy number was unchanged, whereas virus transcription was markedly reduced. While no attempt was made to detect the complete VZV transcriptome, IE63 transcripts were always detected, IE62 transcripts were occasionally detected, and gB transcripts were never detected. Overall, the SCID mouse model provides a useful system to study VZV-human neuronal interactions in which infectious virus is not produced, virus transcription is limited, and VZV DNA is stably maintained.

## 5. Human Neuroblastoma Cells

Neuroblastoma cell lines are derived from human sympathetic nervous system tumors, which often originate in nerve tissues in the abdomen, chest and neck. Differentiation of the neuroblastoma cell line SH-SY5Y into neurons that contain at least one neurite-like projection when examined by phase-contrast microscopy results in cultures that are ~70% neuronal; most neuronal cells with projections stain positive for the neuronal marker synaptophysin [25]. Infection of the differentiated neuroblastoma cells with either cell-free or cell-associated VZV resulted in a CPE, likely because ~30% of the cells are undifferentiated, non-neuronal cells that become productively infected with VZV. Since neuroblastoma cells are not >90% pure neurons, their value in studying the VZV-neuronal relationship is limited to studying neurotropism and productive virus infection. 

## 6. Human Stem Cells

Human embryonic stem cells (hESCs) are derived from the inner cell mass of *in vitro-*fertilized human embryos at the blastocyst stage (first week of development) and can be maintained undifferentiated for at least 8 months (32 passages) in culture [26]. hESCs can differentiate into neurons, a process requiring 30 days in culture and resulting in a population that is ~95% positive for the neuronal marker βIII-tubulin. Importantly, ~10% of the neurons stain positive for both Brn3a and perpherin (markers of sensory neurons) [27]. Interestingly, undifferentiated hESCs and neural progenitor cells (an intermediate stage between undifferentiated and fully differentiated hESCs) are refractory to VZV infection [28]. However, fully differentiated hESC-derived neurons infected with cell-associated VZV develop a CPE within 1–3 weeks, with release of virus into the tissue culture medium. 

hESC-derived neurons can be infected transaxonally, as evidenced by infection of neurons maintained in microfluidic chambers. These chambers contain two culture wells joined by 8- to 12-μm channels. Neurons are seeded into one well of the chamber, and medium with excess nerve growth factor (NGF) is placed into the second chamber, which stimulates axonal growth through the channels towards the chamber with the greater NGF concentration [27,29]. Addition of cell-associated VZV to the second chamber containing axons in high NGF medium results in neuronal infection that spreads throughout the first chamber, indicating transaxonal infection of hESC neurons. 

Human neuronal stem cells (hNSC) derived from fetal brain at 9 weeks’ gestation have also been used to study the VZV-neuronal relationship. Infection of hNSCs that were ~90% neuronal (as revealed by MAP2a and β-tubulin staining) with cell-free VZV (Zostavax vaccine) at a low MOI (~0.0025) revealed no CPE, and viral DNA, transcripts and protein (gE) were found 2 weeks later [30]. Transfer of either the tissue culture supernatant or a homogenate of the infected neurons onto permissive fibroblasts failed to produce a CPE, indicating a non-productive neuronal infection. The differing findings in VZV-infected hESCs and hNSCs likely reflect the MOI and not the cell type or mode of VZV infection. Low MOI of hESCs results in non-productive neuronal infection, while high MOI leads to productive infection [31].

## 7. Induced Human Pluripotent Stem Cells (iPSC)

Induced pluripotent stem cells (iPSCs) are fibroblasts that are de-differentiated to mimic embryonic stem cells and then induced to differentiate into neuronal cells. This process requires at least 2 months *in vitro* and yields cells that are ~80% βIII-tubulin-positive, a minority of which are also positive for the Brn3a and peripherin markers of sensory neurons [32]. After infection with cell-free VZV, a CPE developed in non-neuronal cells, but not in Brn3a/perpherin-double-positive cells, although they were positive for VZV IE62 and gE. The tissue culture medium from the VZV-infected iPSCs produced a CPE in uninfected fibroblasts, possibly reflecting productive infection of the ~20% non-neuronal cells.

**Figure 1:**
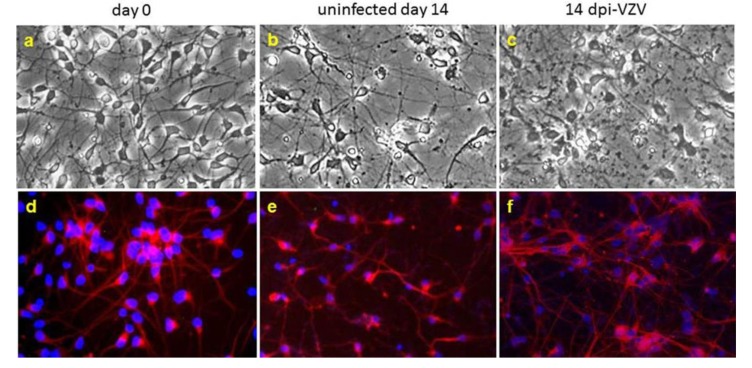
Varicella zoster virus (VZV) infection of highly pure human neurons does not produce a cytopathic effect (CPE). Phase-contrast microscopy of terminally differentiated neurons maintained in tissue culture for up to 21 days showed healthy-appearing neurons on day 0 (**a**) and day 14 in culture (**b**) as well as 14 days after VZV infection (**c**). Immunofluorescence staining (**d**–**f**) with anti-βIII-tubulin antibody revealed positive staining for the neuronal marker (red). Nuclei stained blue with DAPI. Copied and modified with permission from J. Neurovirol [33].

iPSCs are also available commercially. iCell neurons (Cellular Dynamics, Madison, WI) are >95% βIII-tubulin-positive. Two weeks after infection with cell-free VZV (Zostavax vaccine), neuronal cultures appear viable, do not exhibit a CPE (Figure 1) or release infectious virus, but do contain VZV DNA, transcripts and protein in the absence of activated apoptotic proteins [33]. 

**Figure 2:**
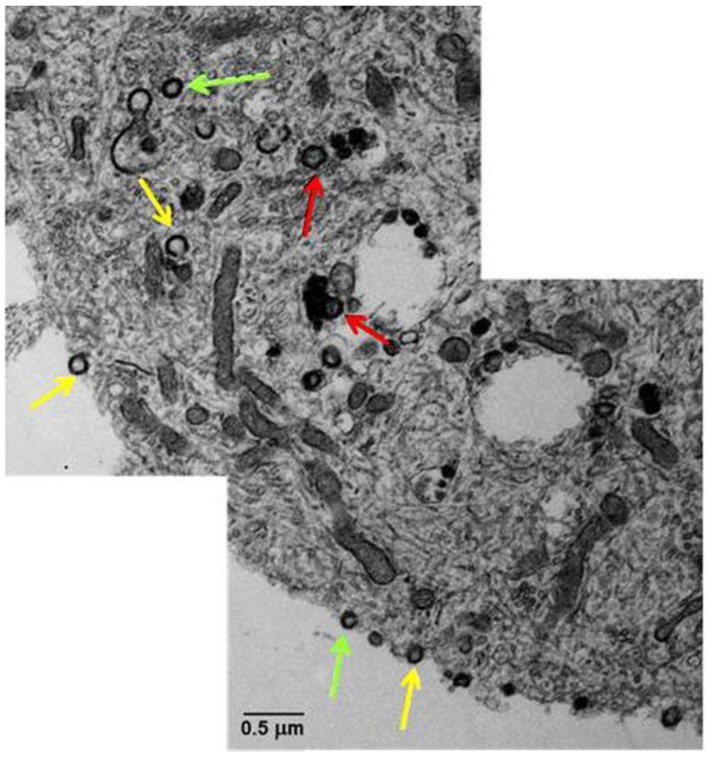
Transmission electron microscopy of VZV-infected human neurons derived from induced pluripotent stem cells. A montage of the cytoplasm and cell surface of a VZV-infected neuron showed viral particles without capsids and viral DNA (yellow arrows), viral particles with capsids but not viral DNA (green arrows), and complete viral particles with capsid and DNA (red arrows). Copied and modified with permission from J. Virol [34].

Ultrastructural analysis of infected iPSCs (Figure 2) shows numerous aberrant virions, most of which are capsids lacking a DNA core or light particles (envelopes without capsids) [34]. 

Quantitative RT-PCR and confocal microscopy show that gC transcripts and protein are greatly diminished in neurons compared to levels in productively infected fibroblasts [34], suggesting that defective virus assembly and diminished virion egress result from low gC expression and account for non-productive VZV infection in neurons. 

**Figure 3:**
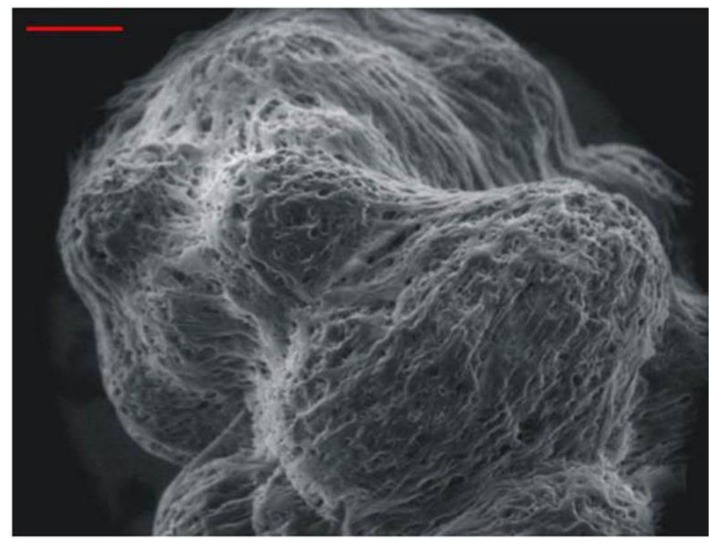
Scanning electron microscopy of 3-dimensional tissue-like assemblies of normal human neuronal progenitor cells maintained for 6 months in suspension. Note the indistinguishable nature of individual cells. Cells assemble around the spherical support matrix, and multiple cell-coated matrixes fuse to form tissue-like assemblies. Bar = 100 mm. Image copied with permission from PLoS Pathogens [35].

## 8. Normal Human Neural Progenitor (NHNP) Tissue-Like Assemblies (TLA)

Human neuronal progenitor cells can be maintained for at least 6 months on inert microspheres kept in suspension within a NASA-designed rotary vessel. Similar to human trigeminal ganglia removed at autopsy [35], these cells form tissue-like assemblies (Figure 3) that express both progenitor and mature neuronal markers. 

Unlike adherent neuron cultures, NHNP-TLAs survive for at least 180 days in culture. Infection with cell-free VZV results in replication of virus DNA and transcription of viral genes for ~18 days, after which a plateau is reached. Importantly, the infected cultures survive for 3 months. Despite their sporadic release of low amounts of infectious virus, NHNP-TLAs provide an opportunity to study the relationship of VZV with human neurons long-term.

## 9. Conclusions

While no *in vitro* system has recapitulated findings in latently infected ganglia, recent studies indicate that VZV infection of neurons *in vitro* can be established in the absence of a cytopathic effect (Table 1), thus holding the promise of molecular analysis of virus-neuronal interactions to discover the mechanisms of virus reactivation from latency. In humans, the virus persists in ganglionic neurons for decades before reactivation, while traditional 2-dimensional neuronal cultures survive for only a few weeks, and even 3-dimensional human neuronal TLAs can only be maintained for some months. Future studies will require highly pure (>90%), terminally differentiated human neurons capable of prolonged survival *in vitro*. While *in vitro* latency has not been established, non-productive models of VZV infection in human neurons will be useful in elucidating the molecular events leading to virus reactivation *in vivo*.

**Table 1:** Current models of non-productive VZV infection of human neurons.

Model	Virus inoculum	Results	Reference
fetal neural stem cells implanted into SCID mouse brain	cell-associated	3 weeks p.i., VZV proteins encoded by ORFs 62, 63 & 47 are detected; VZV gE is rarely detected.	[23]
fetal DRG implanted under SCID mouse kidney capsule	cell-associated	8 weeks p.i., no infectious virus is released; VZV DNA copy number is stable with limited VZV transcription (62/63 only).	[24]
embryonic neural stem cell (hNSC)	cell-free	14 days p.i., no CPE or release of infectious virus, although VZV DNA, RNA, and protein are detectable.	[30]
induced pluripotent stem cell	cell-free	14 days p.i., no CPE or release of infectious virus, although VZV DNA, RNA, & proteins are detected; virions seen - mostly aberrant; no markers of activated apoptotic pathway present.	[33,34]
human neural progenitor cells tissue like assemblies	cell-free	first 18 days p.i. show an increase of both VZV DNA and RNA, which plateaus. At 3 months p.i., no CPE is evident and only sporadic release of infectious virus is seen.	[35]
